# Corridor quality buffers extinction under extreme droughts in experimental metapopulations

**DOI:** 10.1002/ece3.10166

**Published:** 2023-06-01

**Authors:** Dongbo Li, Jane Memmott, Christopher F. Clements

**Affiliations:** ^1^ School of Biological Sciences University of Bristol Bristol UK

**Keywords:** climate change, drought severity, ecological traps, environmental stressors, habitat fragmentation, perturbations, wildlife corridors

## Abstract

Corridors with good‐quality habitats maintain the spatial dynamics of metapopulations by promoting dispersal between habitat patches, potentially buffering populations, and communities against continued global change. However, this function is threatened by habitats becoming increasingly fragmented, and habitat matrices becoming increasingly inhospitable, potentially reducing the resilience and persistence of populations. Yet, we lack a clear understanding of how reduced corridor quality interacts with rates of environmental change to destabilize populations. Using laboratory microcosms containing metapopulations of the Collembola *Folsomia candida*, we investigate the impact of corridor quality on metapopulation persistence under a range of simulated droughts, a key stressor for this species. We manipulated both drought severity and the number of patches affected by drought across landscapes connected by either good‐ or poor‐quality corridors. We measured the time of metapopulation extinction, the maximum rate of metapopulation decline, and the variability of abundance among patches as criteria to evaluate the persistence ability of metapopulations. We show that while drought severity negatively influenced the time of metapopulation extinction and the increase in drought patches caused metapopulation decline, these results were mitigated by good‐quality corridors, which increased metapopulation persistence time and decreased both how fast metapopulations declined and the interpatch variability in abundances. Our results suggest that enhancing corridor quality can increase the persistence of metapopulations, increasing the time available for conservation actions to take effect, and/or for species to adapt or move in the face of continued stress. Given that fragmentation increases the isolation of habitats, improving the quality of habitat corridors may provide a useful strategy to enhance the resistance of spatially structured populations.

## Graphical abstract

Corridors maintain the spatial dynamics of metapopulations by promoting dispersal between habitat patches, potentially buffering populations and communities against continued global change. Using a lab microcosm experiment, we demonstrate how corridor quality maintains the persistence of metapopulations in the face of extreme climate change.

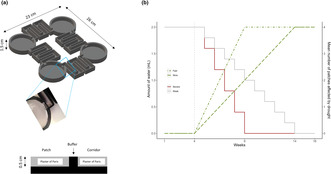

## 1 INTRODUCTION

While droughts are natural phenomenon, their frequency and severity are both increasing due to climate change (Mukherjee et al., 2018), driven by reduced regional precipitation and increased global evaporation (Ault, 2020; Dai, 2011). Drought has devastating impacts on species and ecosystems, causing an increase in species mortality and extinction (Choat et al., 2018; Harrison, 2000), loss of biodiversity (Peterson et al., 2021; Tilman & Elhaddi, 1992), and declines in ecosystem function and productivity (Atkinson et al., 2014; Ciais et al., 2005).

These pressures are occurring against a backdrop of increasing habitat fragmentation, driven by a range of factors including the extraction of resources, development of settlements, increasing transport links, and proliferation of farming globally. Consequently, many populations are increasingly isolated, impacting their risk of extinction (Crooks et al., 2017; Reed, 2004). Corridors have been suggested as positive conservation actions, which can be used to reverse the negative effect of fragmentation by promoting dispersal and maintaining gene flow, allowing species to move or adapt in the face of environmental change. Previous studies have shown that enhancing habitat connectivity by corridors can reduce the likelihood of extinction and enhance species diversity in patchy habitats (Chisholm et al., 2011; Damschen et al., 2019), potentially buffering species loss against increasingly inhospitable environments.

Extreme climatic events such as droughts have a devasting impact on the viability of natural populations. Previous studies showed that droughts reduced the survival rate of populations in habitat patches by inducing ecological traps, a phenomenon that occurs when species settle in maladaptive habitats due to poor habitat selection (Hale & Swearer, 2016; Robertson & Hutto, 2006). For example, Coho salmons (*Oncorhynchus kisutch*) that inhabit intermittent streams used remaining pools as habitats when connectivity was lost, but during drought years a lower survival rate was found in some pools than in others (Vander Vorste et al., 2020). Such phenomena have also been reported in other ecosystems (Robertson & Hutto, 2006). In this case, dispersal might be key to maintain the dynamics of metapopulations and communities (Hale et al., 2015; Hale & Swearer, 2016; Wolfe et al., 2023), as climatic extremes are likely to occur dynamically across time and space, and the quality of habitats can deteriorate or recover when the climate regime changes. Yet, there is relatively few data on the population changes with temporarily and spatially dynamic drought stressors.

Corridor quality is one of the important physical properties determining corridor effectiveness and dispersal success (Bennett et al., 1994; Habel et al., 2020; Li et al., 2021). Specifically, good‐quality corridors can promote dispersal, leading to greater movement rates to colonized patches compared to poor‐quality corridors (Li et al., 2021). What determines the quality of a corridor is likely specific to the species using that corridor, but will reflect the hostility of a corridor to individuals surviving within it or the ease in which they can pass along it (Haddad & Tewksbury, 2005; Li et al., 2021). Thus, corridor quality is better when corridors contain shelter/resources/water, serving as conduits and habitats, while quality is poorer if they expose individuals to, for example, predation or metabolic strain (Haddad & Tewksbury, 2005). Corridor quality thus becomes increasingly important as the length of a corridor increases and may affect the stability and longevity of corridor‐connected metapopulations or metacommunities by facilitating or impeding movements between habitat patches. However, we currently lack empirical evidence on how corridor quality promotes metapopulation persistence, and how it interacts with the increasing severity and frequency of drought stressors.

While historical data have successfully investigated the population consequences of extreme drought events on habitat networks (e.g., Oliver et al., 2013), quantitively measuring the effect of increasing drought severity and frequency on population declines and – importantly – how this stress might interact with corridors is difficult. Experimental microcosms provide one alternative method to achieve this, as they allow landscape‐style manipulations at an observable scale (Altermatt et al., 2015; Benton et al., 2007). Indeed, such systems have previously been used to study the effect of network modularity (e.g., Gilarranz et al., 2017), and the impact of habitat configuration on metacommunities (e.g., Chisholm et al., 2011; Wolfe et al., 2022).

Here, we investigate the effect of corridor quality on the persistence of metapopulations under drought stressors. We manipulated both drought severity and the number of patches affected by drought in landscapes connected by either good‐ or poor‐quality corridors. We use 3D‐printed four‐patch and four‐corridor microcosms containing the soil Collembola *Folsomia candida* as experimental metapopulations and use a fully factorial manipulation where we change the moisture of corridors (good‐ vs. poor‐quality corridors), amount of water added to the patches (severity of drought), and number of patches (from 1 to 4) affected by reduced water availability (increase in drought patches). Drought is a known abiotic stressor to *F. candida* populations, as they need a high moisture to survive and reproduce. By monitoring the changes in metapopulation abundance in microcosms for a relatively long‐period (*c*. 16 weeks, ~5 generations), we can measure the effects of corridor quality interacting with drought severity and increase in drought patches at the time of extinction, the maximum rate of metapopulation decline, and the variability of abundance among patches.

## 2 METHODS

### 2.1 Model organism

The model organism is the soil arthropod *Folsomia candida* (Collembola, Isotomidae). *F. candida* was reared in Petri dishes at room temperature (*c*. 19°C), with dry yeast provided as a food resource. Although *F. candida* can survive in drought conditions for short periods by temporarily changing their physiological response (Fountain & Hopkin, 2005), humidity is essential to their lifecycle (Waagner et al., 2011). Laboratory studies have shown that both the survival rate and egg production of *F. candida* were decreased when air humidity reduced from 100% to 96% RH (Hilligsoe & Holmstrup, 2003; Waagner et al., 2011). *F. candida* eggs are more sensitive to drought and will perish if relative humidity below 98.7% (Holmstrup, 2019).

### 2.2 Experimental landscapes

**FIGURE 1:**
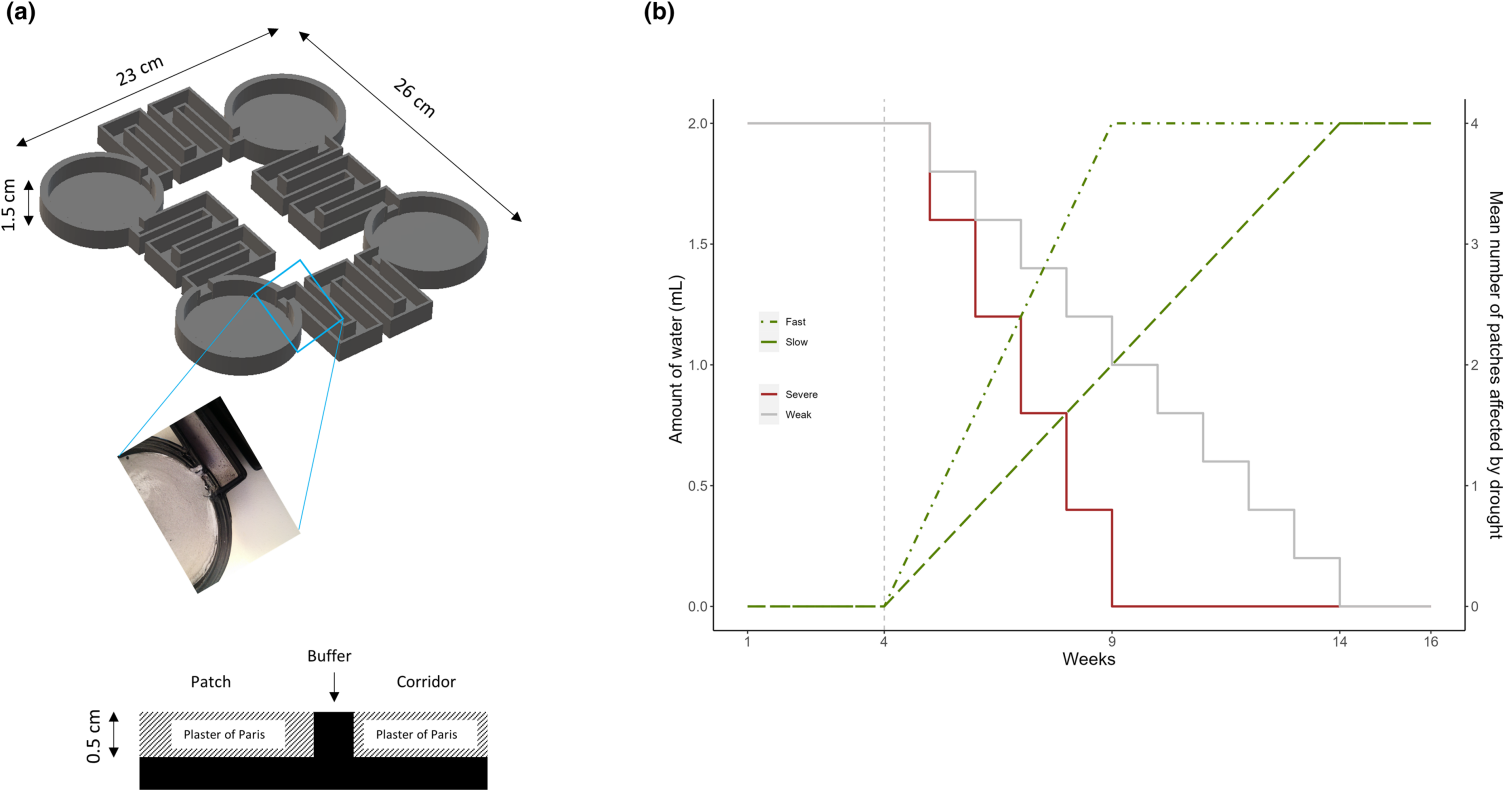
(a) The four‐patch microcosm; inset and diagram show the portion of a microcosm complete with a Plaster of Paris fill (0.5 cm thickness) and the patch‐corridor plastic buffer (which prevents moisture moving between the two via the Plaster of Paris); (b) The quantity of water added to the microcosms over time and the ensuing number of patches affected by drought. The microcosms experienced drought severity and increase in drought patches, after treatments start at week 4 (gray dashed line). The number of dry patches in the fast increase in drought patches (green dot‐dashed line) treatment reaches 4 at week 9, while in the slow (green long‐dash line) reaches 4 at week 14. Severe drought is created by reducing the amount of water 0.4 mL per week (brown solid line), while weak drought is created by reducing water at 0.2 mL per week (gray solid line).

The 3D‐printed experimental arenas were made from PLA plastic (3D printer: LulzBot TAZ 6; filament: Winkle PLA 2.85 mm, Spain) and consisted of four habitats patches (circular: *c*. 7 cm in diameter and 1.5 cm in height) connected by four corridors (*c*. 28 cm in length and 1 cm in width) in a square (2 × 2) configuration, with each habitat patch connected by two corridors. Both habitat patches and corridors were filled with a mixture of plaster of Paris and black dye (*c*. 0.5 cm in thickness). Plaster of Paris acts as a substrate for the Collembola to live on which keeps moisture, while the black dye makes Collembola more visible for counting (Li et al., 2021). Our pilot work showed that moisture could wick between the different components of the landscapes (patches and corridors), thus—to avoid this—plastic baffles (0.5 cm in height) were printed allowing the moisture of the corridors to be manipulated separately to the moisture of the patches (Figure 1a).

**FIGURE 2:**
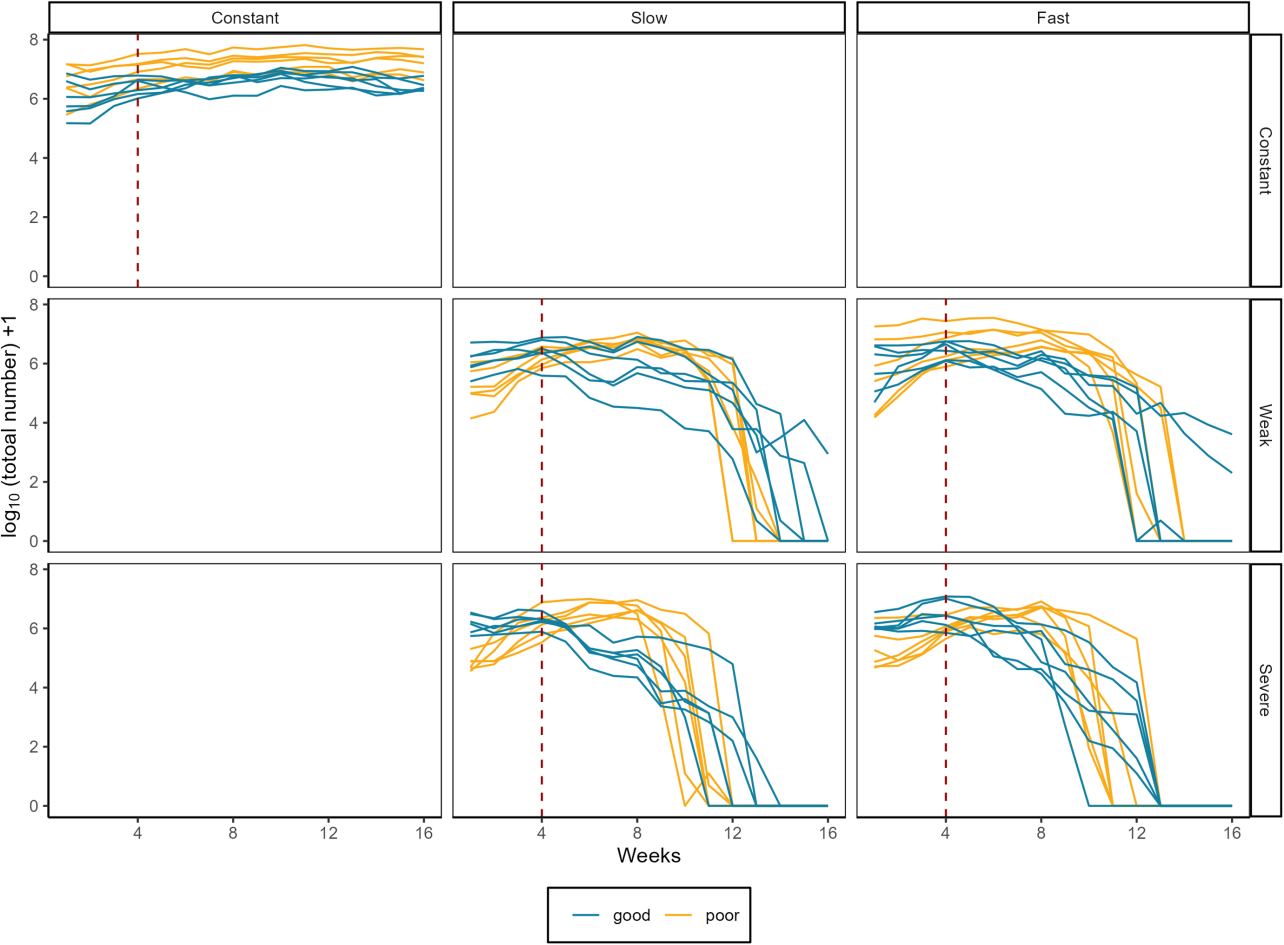
The total number of individuals in each microcosm changes over time, from the beginning (week 1) to the end of experiment (week 16). Drought treatment starts at week 4 (red dashed line). Constant (control) treatments are shown at the top left panel, and the four panels at bottom right show the combinations of drought treatments, that is, drought severity (weak vs. severe) and increase in drought patches (slow vs. fast). Good‐quality corridor microcosms are shown in blue, while poor‐quality corridor microcosms are shown in yellow. Data is log (*x* + 1) transformed; number of replicates for each treatment *N* = 6. Stable lines in two constant treatments indicate that populations were not stressed and remained stable throughout the experiment. The potential confounding effect mentioned in the methods section can be seen in the differing starting populations across the treatments.

As Collembola are delicate and not easy to handle, they were inoculated into the four patches of each landscape by tapping individuals (mean ± SD = 423 ± 317) into each microcosm (week 1 of the experiment). During the experimental setup, a potential confounding effect was accidentally introduced whereby microcosms were not randomly assigned to treatment after introducing the Collembola. This may have caused systemic differences in population‐level responses to treatments (Figure 2), which we have attempted to account for by including microcosm as a random effect in the statistical models where appropriate (see below). We fed the population in each patch each week with a piece of dry yeast flakes (7 ± 2 mg mean ± SD, number of measures *n* = 30) throughout the experiment, and thus food was not a limiting factor to growth. Collembola were reared in the microcosms for 3 weeks prior to the start of treatments to allow metapopulations to stabilize, with 2 mL water and 7 mg of food added each week to each patch. Clear lids were used to cover the microcosms and stop them drying out too quickly, and all microcosms were placed in dark rearing room to minimize light exposure.

### 2.3 Treatments

We used a fully factorial experiment to investigate the interactive effects of corridor quality with drought severity and increase in drought patches across landscape on the persistence of metapopulations. In the good corridor quality treatment, corridors had 2 mL of water added each week to keep them moist (Li et al., 2021), while in the poor corridor quality treatment no water was added to the corridors. For each corridor quality treatment (i.e., good or poor), we simultaneously manipulated drought in two different ways: changing the rate at which the moisture content of patches declined (i.e., drought severity), and increasing the number of patches simultaneously experiencing drought (i.e., increase in drought patches). There were two levels of drought severity (i.e., severe vs. weak), and within each severity level, there were two levels of increasing drought patches (i.e., fast vs. slow). Severe drought was created by reducing the 2 mL of water added to each patch by 0.4 mL water per week, while weak drought was simulated by reducing the 2 mL by 0.2 mL water per week (Figure 1b). In addition, for the “increase in drought patches” treatment we randomly selected a number of patches to not receive water in any given week. This number was increased through time and determined by drawing a number (from 0 to 4) from a binomial distribution with the probability increasing from 0 to 1 at either 0.2 per week (i.e., fast) or 0.1 per week (i.e., slow; Figure 1b). This was done for each replicate landscape, and the location of patches impacted by this was randomized for each landscape. As such, all the habitat patches within a landscape were manipulated with a reduced amount of water per week (i.e., drought severity), unless any of the patches were randomly assigned not receiving water (i.e., increase in drought patches). We also implemented a constant (control) treatment for each corridor quality group (i.e., good or poor) where 2 mL water were constantly added to all the patches each week. This gave us in total two levels of corridor quality (good vs. poor), two levels of drought severity (severe vs. weak), two levels of increase in the number of drought patches (fast vs. slow), and one control group (no change in water through time), with each treatment combination replicated six times for a total of 60 replicates. The experiments lasted 16 weeks total, with the drought treatments started at week 4. All the microcosms were monitored weekly until the metapopulations in drought treatments went extinct.

### 2.4 Metapopulation monitoring

Metapopulations in the landscapes were monitored by taking three photos in quick succession of each habitat patch once every week. These pictures were then used to count individuals within each patch using Fiji software (Schindelin et al., 2012), a background subtraction method to determine which individuals are alive by comparing, which have moved between the three photos (Mallard et al., 2013). Due to limitation in the quality of the photos taken, we were only able to count individuals larger than 0.2 mm in length, equal approximately to the size of individuals at eclosion (Johnson & Wellington, 1983).

### 2.5 Data analysis

All the statistics were conducted in R (v4.0.2, R Core Team, 2022). We assessed how corridor quality, drought severity, and an increase in drought patches influence the persistence ability of metapopulations in three ways. First, we investigated how those factors influence the time of metapopulation extinction. The time of metapopulation extinction was defined as the midpoint between the last week, which individuals were observed alive in at least one of the four patches of a landscape, and the first time no individuals were observed as being alive. Only three microcosms were alive at the end of the experiment (week 16, Figure 2), but no individuals were observed alive the week after that, thus the time of extinction for these three microcosms were recorded as week 16.5. As there was only one time of metapopulation extinction for each microcosm, we could not use microcosms as a random effect in this analysis. Instead, we used a generalized linear model (GLM) with a log‐link Gaussian error to fit these data. Experimental factors (i.e., corridor quality, drought severity, increase in drought patches) and all their two‐way interactions were included as explanatory variables. As the metapopulations in the constant (control) treatments were stable (and thus we did not observe any extinctions (Figure 2)) we removed these from the time of extinction analysis and the maximum rate of decline analysis (see below).

Second, we used a generalized linear mixed effect model (GLMM) with a zero‐inflated negative binomial distribution to investigate how corridor quality, drought severity, and increase in drought patches influence the variability of abundance among patches through time. The variability of abundance among patches was defined as the standard deviation of abundances between the four habitat patches. Microcosm identity was included as a random effect to account for the potential differences between microcosms including their starting densities. We included time, corridor quality, drought severity, increase in drought patches, and their interactions as fixed effects. Of particular interest was how experimental factors (i.e., corridor quality, drought severity, increase in drought patches) and time interactively affect the changes in variability, thus we also include their two‐way interactions in the fixed effect. Time and experimental factors (i.e., corridor quality, drought severity, increase in drought patches) were included as a zero‐inflation parameter to account for the overdispersion of zero counts after extinction occurred. Data collected before drought treatment started (i.e., from week 1 to 3) was excluded from model fitting. The model was fitted using “glmmTMB” package (Magnusson et al., 2017).

Finally, we used nonlinear regression curves with a three‐parameter logistic function to fit the changes in total population abundance in each habitat network over time. Microcosms either in good‐ or poor‐quality corridor treatment experienced the same rearing conditions prior to the start of the drought treatments (i.e., week 1–3), thus we only fit the nonlinear regression curves with the data on metapopulation abundance from week 4 to week 16 (Appendix S1). The model was specified as *Y* = *d*/(1 + exp (−b*(*X*−*e*))) where *Y* was total abundance and *X* was time; *d* was the high asymptote; *b* was the maximum slope; *e* was the time at the maximum slope, were all estimated by models. This is a three‐parameter logistic function, and we chose this model due to its simplicity and utility (Kingsland, 1982). Models were fitted using “drc” package (Ritz et al., 2015). To explore the effect of treatments on the maximum rate of metapopulation change, we extracted the estimated value of *b* in each model and compared across treatments (Figure S1). Again, as there is only one estimated value of the rate of metapopulation change (i.e., *b* parameter) for each microcosm, we used a GLM with a Gaussian distribution to fit the log‐transformed data, where corridor quality, drought severity, increase in drought patches, and their two‐way interactions were included as explanatory variables.

## 3 RESULTS

**FIGURE 3:**
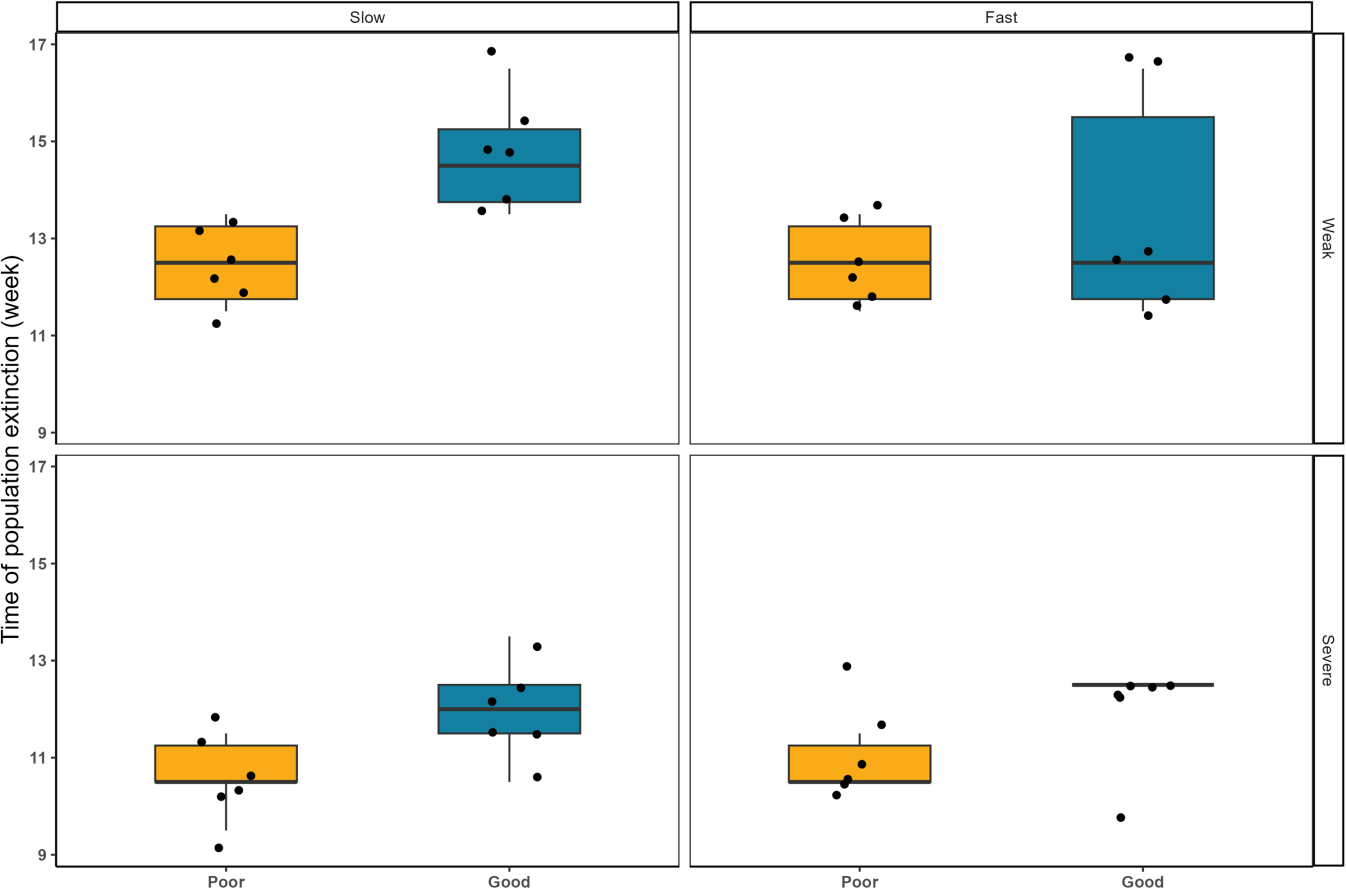
The effect of corridor quality, drought severity, and increase in drought patches on the time of population extinction. Each panel shows the time of metapopulation extinction (weeks) in microcosms connected by either poor or good‐quality corridors, under specific combination of drought severity (weak vs. severe) and increase in drought patches (slow vs. fast). Data is shown as black dots. The maximum and minimum limits are shown by the end of whiskers, and the first and third quartiles of responses are shown by the end of boxplots. The lines inside the boxplots represent the medians. Number of observations *N* = 6.

As expected, the fitted model (pseudo *R* ^2^ = .527) showed that drought severity negatively affected the time of metapopulation extinction (Table 1, Figure 3). The increase in drought patches, however, had no effect (Table 1). Corridor quality positively affected the time of metapopulation extinction (Table 1), indicating that metapopulations persisted longer in habitats connected by good‐quality corridors. There was no effect of any of the two‐way interactions of treatments on the time of metapopulation extinction (Table 1).

**TABLE 1:** The effect of corridor quality, drought severity, and increase in drought patches on the time of population extinction.

	Estimate	Stand error	*t*‐Value	*p*
Intercept	2.532	0.038	67.258	<.001***
Corridor quality	0.149	0.047	3.163	.003**
Drought severity	−0.173	0.053	−3.289	.002**
Increase in drought patches (IP)	−0.012	0.050	−0.249	.805
Corridor quality: drought severity	−0.016	0.058	−0.276	.784
Corridor quality: IP	−0.061	0.058	−1.050	.300
Drought severity: IP	0.060	0.058	1.026	.311

*Note*: Significant levels: “***” .001 “**” .01 “*” .05.

**FIGURE 4:**
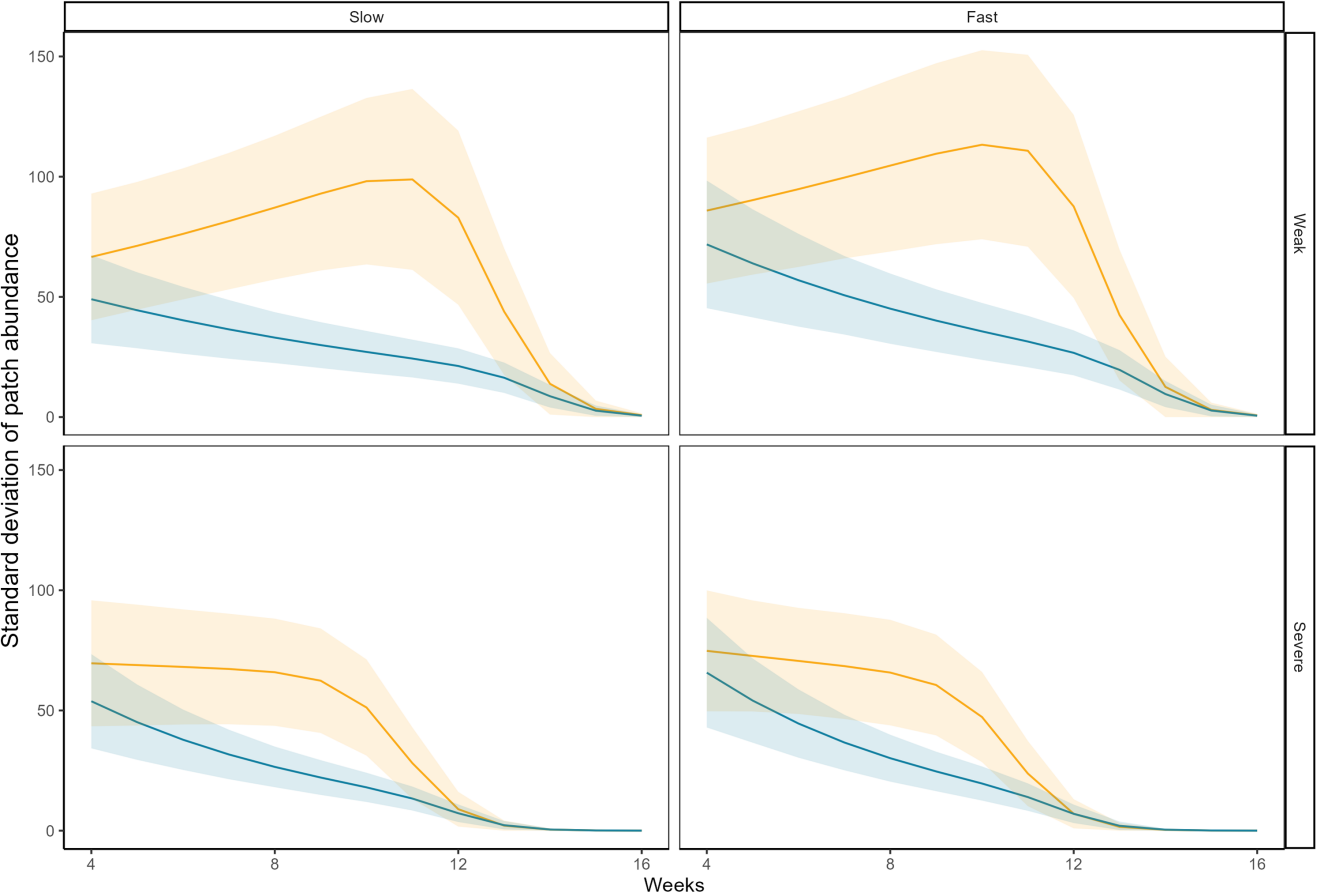
Results from zero‐inflated GLMM with a negative binomial distribution show the effect of corridor quality, drought severity, and increase in drought patches on the standard deviation of patch abundance from week 4 to week 16. Each panel shows the changes in the standard deviation of patch abundance over time in landscapes connected either by good or poor‐quality corridors under specific combination of drought severity (weak vs. severe) and increase in drought patches (slow vs. fast). Good corridor quality treatment is shown in yellow and poor corridor quality treatment is shown in blue, with 95% confidence intervals (CIs).

Our fitted model (conditional/marginal *R* ^2^ = .417/.336) showed that time negatively affected the variability of patch abundance (Table 2), suggesting an overall decrease in variability over time (Figure 4). The variability of patch abundance was negatively affected by corridor quality, with higher variability found in landscapes connected with poor‐quality corridors. However, this was not affected by drought severity and increase in drought patches (Table 2). There were significant interactions between time and corridor quality, and between time and drought severity (Table 2), suggesting that the effect of corridor quality, as well as drought severity, was largely time‐dependent. Interestingly, the different changes in variability over time between good and poor corridor quality suggest a contrasting response of metapopulations to stressors due different levels of connectivity. In habitats with poor‐quality corridors, the rate of decrease in variability of patch abundance aggravated by continual drought stress, while in those with good corridor quality the rate of decrease in variability eased (Figure 4). There were no significant interactions between other two‐way factors (Table 2).

**TABLE 2:** The effect of corridor quality, drought severity, and increase in drought patches on the variability of patch abundance.

	Estimate	Stand error	*z*‐Value	*p*
(Intercept)	4.963	0.242	20.546	<.001***
Time	−0.194	0.024	−8.191	<.001***
Corridor quality	−0.534	0.272	−1.961	.050.
Drought severity	−0.223	0.283	−0.788	.430
Increase in drought patches (IP)	−0.271	0.273	−0.991	.321
Corridor quality: Drought severity	0.049	0.249	0.198	.843
Corridor quality: IP	0.128	0.248	0.515	.606
Drought severity: IP	−0.183	0.249	−0.733	.463
Time: Corridor quality	0.166	0.022	7.382	<.001***
Time: Drought severity	0.078	0.023	3.384	<.001***
Time: IP	0.018	0.022	0.814	.415

*Note*: Significant levels: “***” .001 “**” .01 “*” .05 “.” .1.

**FIGURE 5:**
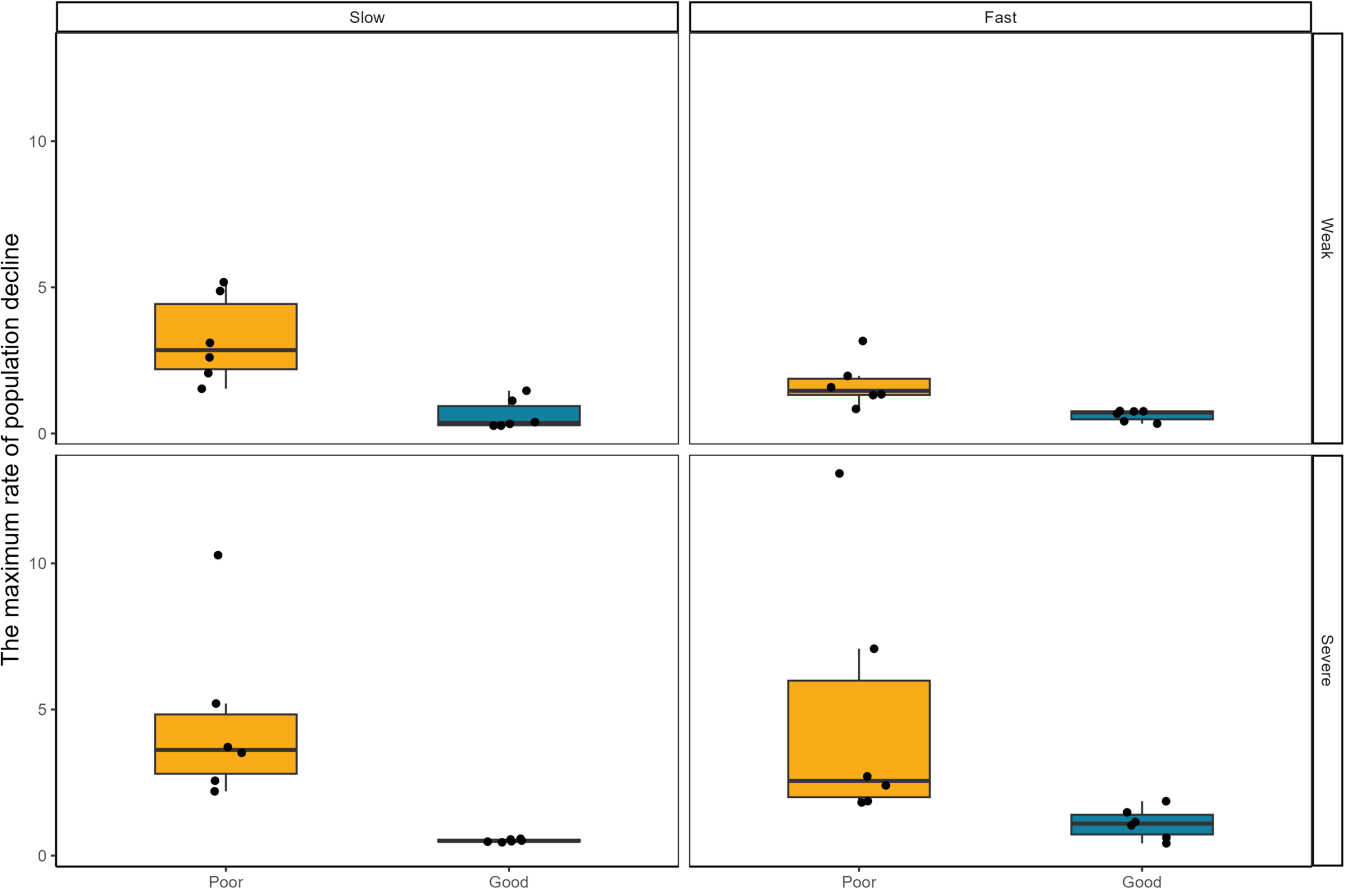
The effect of corridor quality, drought severity, and increase in drought patches affected on the maximum rate of population decline. For annotations, see the legend for Figure 3.

The results of our fitted GLM model (pseudo *R* ^2^ = .732) showed that corridor quality negatively affected the maximum rate of metapopulation decline, with their declining rate being slower in good than poor corridor quality treatment (Table 3, Figure 5). Surprisingly, an increase in the number of drought patches negatively affected the rate of metapopulation decline, but drought severity had no effect (Table 3, Figure 5). Corridor quality positively interacted with the increase in drought patches (Table 3). There were no effects of other two‐way interactions on the maximum rate of metapopulation decline (Table 3).

**TABLE 3:** The effect of corridor quality, drought severity, and increase drought patches on the maximum rate of population decline.

	Estimate	Standard error	*z*‐Value	*p*
Intercept	1.072	0.207	5.187	<.001***
Corridor quality	−1.767	0.271	−6.534	<.001***
Drought severity	0.314	0.271	1.161	.252
Increase in drought patches (IP)	−0.620	0.271	−2.291	.027*
Corridor quality: Drought severity	−0.297	0.312	−0.951	.347
Corridor quality: IP	0.781	0.312	2.501	.017*
Drought severity: IP	0.499	0.312	1.596	.118.

*Note*: Significant levels: “***” .001 “**” .01 “*” .05 “.” .1.

## 4 DISCUSSION

Our results show that, in our experimental microcosms, increased corridor quality can buffer a metapopulation from extinction caused by drought. Specifically, habitats connected by good‐quality corridors, not only enabled metapopulations to persist longer when they are threatened by drought severity, but slowed down the rate at which the increase in drought patches decreased metapopulation abundance. Surprisingly, corridor quality differently affected the survival of population in each individual patch, as the variability of patch abundance increased or slightly decrease then followed by a sudden decrease in the habitats connected by poor‐quality corridors, while it constantly decreased in the good‐quality corridors. In what follows, we first discuss the limitations of our approach and then discuss the results in the context of the literature.

There are two main limitations to our approach. The first is that we did not count individuals in the corridors, as corridors can, potentially at least, provide extra habitats and serve as temporal refugia for species when stressed (Eversham & Telfer, 1994). The aim of the work was to investigate how patchy populations response to environmental stressors, thus the individuals dispersing in corridors are largely ignored. The second is the potential confounding effect introduced the setup of the experiment, which may explain some of the observed patterns in the abundance dynamics between treatments (Figure 2). This may be due to systematic differences in the body size distributions of the populations in different treatments; however, the extinction dynamics largely conform to the expected patterns.

Species are expected to seek new habitats when natal habitats deteriorate. Field studies showed that Collembola have a high dispersal ability for new habitats when experiencing dry conditions (e.g., Ferrin et al., 2022). Consequently, increasing movement between habitats may save populations from local extinction when patches experience inhospitable environmental conditions. Our previous work showed that Collembola were more likely to colonize new habitats connected by good‐quality corridors, thereby increasing population growth rate in the colonized patches (Li et al., 2021). In this regard, good‐quality corridors may increase their probability of survival by facilitating movements and avoiding dry habitats. Collectively, we demonstrated that good‐quality corridors reduced the rate of population decline and increased time before extinction caused by drought extremes, highlighting the importance of corridors with good habitats in persisting metapopulations in the face of climate change. This not only agrees with previous studies, which show that increasing connectivity using good‐quality corridors can increase the persistence of metapopulations (Gonzalez et al., 1998; Swart & Lawes, 1996), but proves their ability to slow down extinction when species are exposed to disastrous environmental conditions. Our results suggest that increasing corridor quality across a landscape can to some extent mitigate the negative impact of climatic extremes on metapopulations.

The most striking results were that the variability of patch abundance was negatively related to increased corridor quality, suggesting that corridor quality can play an important role in shaping the viability of patchy populations. Thus, when corridor quality was poor, Collembola were constrained in patches as their dispersal ability was limited. Intriguingly, this implies that when corridor quality is good the increased migration of individuals around the landscape could put increased pressure on habitats to support large metapopulation sizes, increasing the impacts of density dependence if such patterns persist in the long‐term. Conversely, individuals being stuck in a patch could cause uneven survival rates among patches when drought extremes occur unpredictably. In such settings, local extinctions were more likely to occur when some patches were turned into ecological traps due to environmental change (Hale & Swearer, 2016), thereby increasing the variability of patch abundance in habitats with poor habitat connectance. Similar findings have been previously reported in freshwater systems where drought disrupted the connectivity of habitat pools for some fish species (e.g., Vander Vorste et al., 2020). Furthermore, the changes in variability of patch abundance over time indicates a contrasting pattern of stressed metapopulations toward extinction between habitats connected with good and poor corridors. Specifically, a constant decrease of the variability found in well‐connected populations indicates that population size became more uniform across patches within metapopulations while experiencing stress, while a large variability found in poor connected populations demonstrated an increasing risk of local extinction because of loss of corridor quality. Our results suggest that the variability of patch abundance may be a good predictor of population status and monitoring how it changes over time might provide a useful guide to evaluate landscape connectivity for species.

Drought is a climatic extreme, which has serious consequences for the persistence of metapopulations, seen here as an increasing drought severity significantly reducing the time that metapopulation persisted and increasing the rate of metapopulation decline, a phenomenon, which is consistent with the prediction that severer drought would cause higher speed of extinction (Cady et al., 2019; Cayuela et al., 2016). Increasing the severity of drought is more likely to cause a strong effect of desiccation, causing a high rate of mortality when reaching species' limits to drought. Collembola are well adapted in humid soil environments and need to absorb water vapor over their entire life cycles (Bayley & Holmstrup, 1999). Some essential life stages such as reproduction cycles and egg incubation in Collembola are highly depended on moisture levels (Holmstrup, 2019; Waagner et al., 2011). Meanwhile, young individuals of Collembola are more vulnerable to desiccation than old (Hilligsoe & Holmstrup, 2003), increasing drought severity are more likely to reduce the fitness of young adults and cause a further loss of fecundity. This may explain the extinction event that occurred at the end of our experiment, and the extinction happens faster with increased severity. Indeed, extreme environmental stressors such as drought have been shown to shape the population dynamics and persistence of many species (e.g., Johansson et al., 2020).

The amount of the favorable habitats within a landscape has been shown to be important for maintaining the persistence of metapopulations (Dytham, 1995; Meli et al., 2014). Surprisingly, we found that increasing the number of patches affected by drought at different speeds only negatively affect the maximum rate of metapopulation decline, rather than the timing of extinctions, which may suggest a tipping point for environmental conditions as a result of increased drought habitats destabilizing populations. The reasons underlying this pattern are largely unexplored though. One possible explanation is that Collembola were able to accumulate sugar and polyols when experiencing dry conditions, temporarily prolonging their survival from dehydration (Fountain & Hopkin, 2005; Waagner et al., 2012). Hence, it is possible that individuals living in a habitat patch under drought manipulation (i.e., no water added for a week in our case) might not experience an immediate local extinction due to the accumulation of polyols and sugars, though drought was shown suppressing the viability of populations based on our results (Figure 2). Adjusting their body conditions may allow them to temporarily survive over a short drought period; however, it may also contribute to a fast decline when the environmental conditions reach to tipping points (Dai et al., 2012). More empirical evidence is needed to examine how increased environmental stress, associated with tipping points, impacts the resilience of spatially structured populations.

In summary, our analyses add to the growing literature describing how increasing corridor quality among habitats can buffer metapopulations against decline and extinction. However, we also show that increased corridor quality may increase the variability of populations between patches in a landscape, as individuals can move more easily into patches with more favorable conditions, potentially increasing the effects of density dependence in these highly utilized patches. As habitat fragmentation creates a mosaic of landscapes hosting a variety of spatially structured populations, maintaining spatial connectivity by good quality of corridors allows species to move out the dangerous area, and/or allows conservation actions to be taken to avoid mass extinctions when facing extreme climatic events. Ultimately, our work highlights the importance of corridor quality in maintaining metapopulation viability in the face of climate change. Field evidence is needed to better understand the role of corridor quality in persisting metapopulations.

## AUTHOR CONTRIBUTIONS

**Dongbo Li:** Data curation (equal); formal analysis (equal); funding acquisition (equal); investigation (equal); methodology (equal); writing – original draft (equal). **Jane Memmott:** Supervision (equal). **Christopher F. Clements:** Supervision (equal).

## CONFLICT OF INTEREST STATEMENT

The authors declare no conflict of interests.

## Supporting information


Appendix S1

## Data Availability

Data are available to access in Dryad (https://doi.org/10.5061/dryad.xsj3tx9mh) and GitHub repository (https://github.com/Dongboli/experimental‐data.git).
