# Supplementary material for: Corridor quality buffers extinction under extreme droughts in experimental metapopulations
Source: Ecol Evol. 2023 Jun 1;13(6):e10166. doi: 10.1002/ece3.10166 (PMC10234780; doi:10.1002/ece3.10166)
Supplement: Supplementary file 1 — Appendix S1 [file ECE3-13-e10166-s001.docx]

**Appendix S1**

**Corridor quality buffer extinction under extreme droughts in experimental metapopulations**

***Contents***

**1. Non-linear regressions**

**2. Figure S1**

**3. Model diagnose**

**5. References**

**1. Non-linear regressions**

We fitted total abundance data with Dose-Response regression curves (Ritz, Baty, Streibig, & Gerhard, 2015). The changes in population abundance over time in each microcosm (from week 4 to 16) was fitted using a three-parameter logistic regression curve which was specified as Y= d / (1+ exp (- b * (X - e))) where Y was total abundance and X was time; d the high asymptote, b the maximum slope and e the time at the maximum slope were estimated by models. Thus, the fitted models in our total 60 microcosms were shown in 60 panels in Fig. S1.


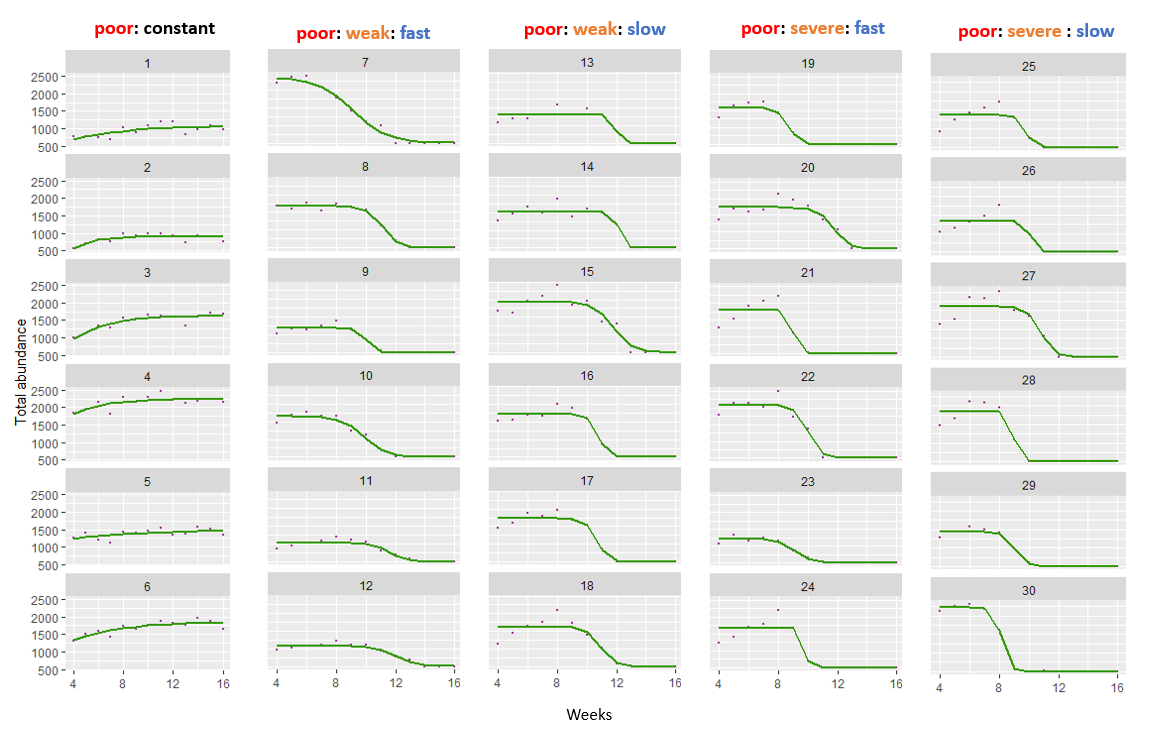


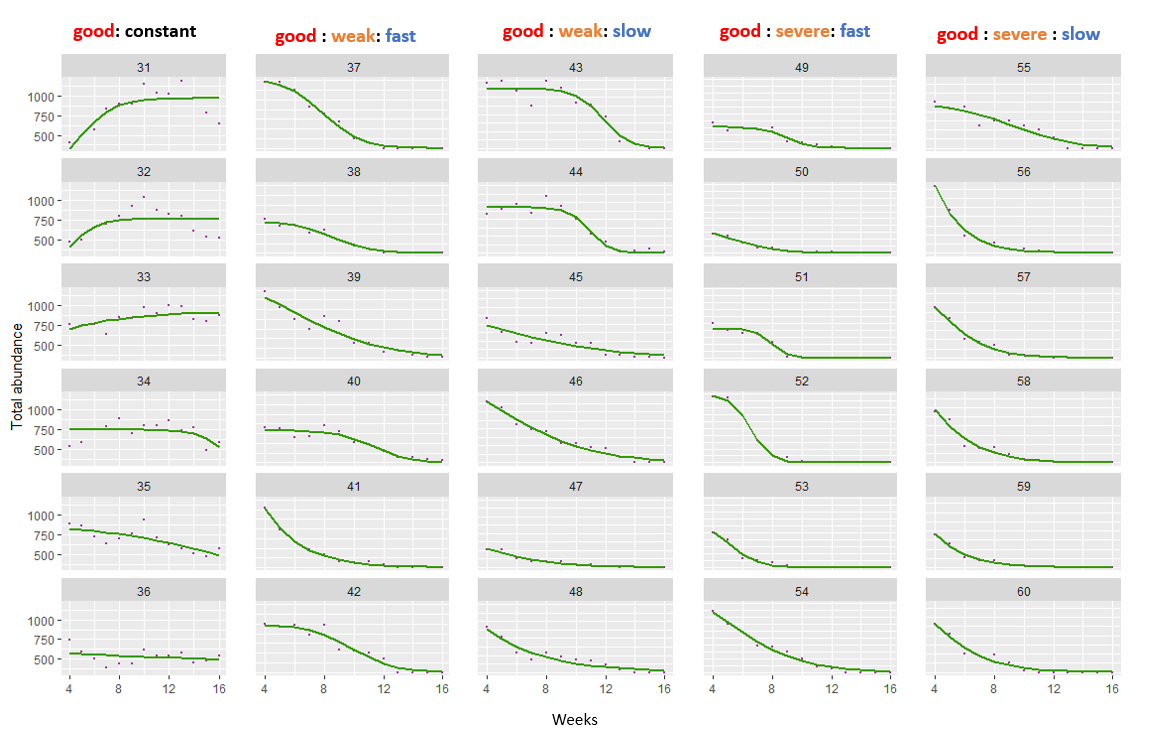


**Figure S1**. The fitted models (green lines) of population abundance over time (purple points) in microcosms under different treatments of corridor quality (good vs. poor), drought severity (severe vs. weak), and increase in drought patches (fast vs. slow). Panel numbers indicate microcosm identities; number of replicates N=6.

**2. Model diagnose**

All models were diagnosed using ‘DHARMa’ package (Hartig & Hartig 2017). Each model creates two panels which show a QQ-plot on the left and a residual plot on the right, based on simulated model residuals. The QQ-plot is used to detect the overall deviation between expected and observed distribution, by adding a Kolmogorov-Smirnov test, a dispersion test, and an outlier test. The residual plot produces DHARMa residuals against either predicted values or predictors.


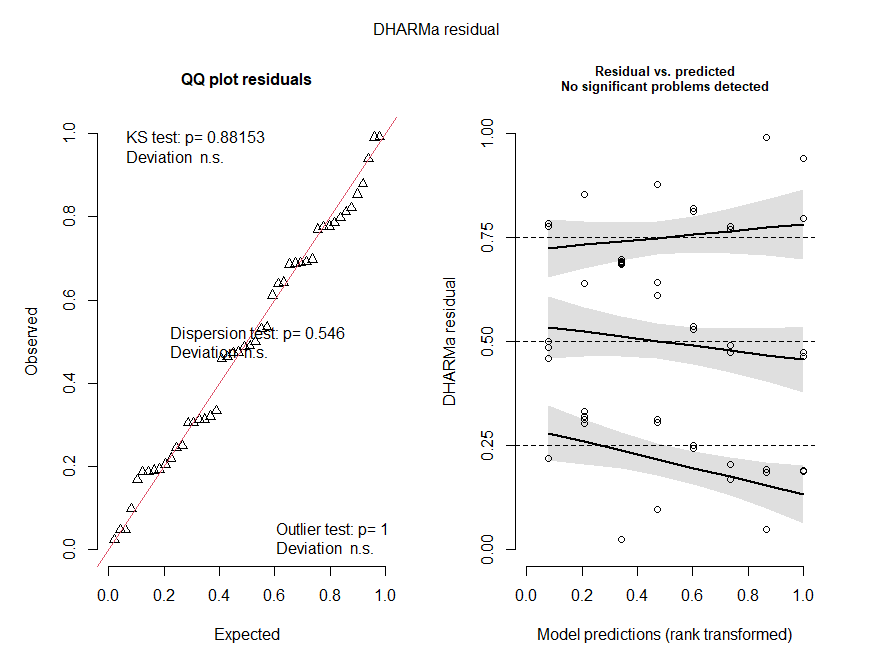


**Figure S2** Residual plots of the Gaussian GLM with a log-link function fitted with data on the time of metapopulation extinction. Both the QQ plot (left) and the residual plot (right) shows that there are no differences between observed and expected residuals.


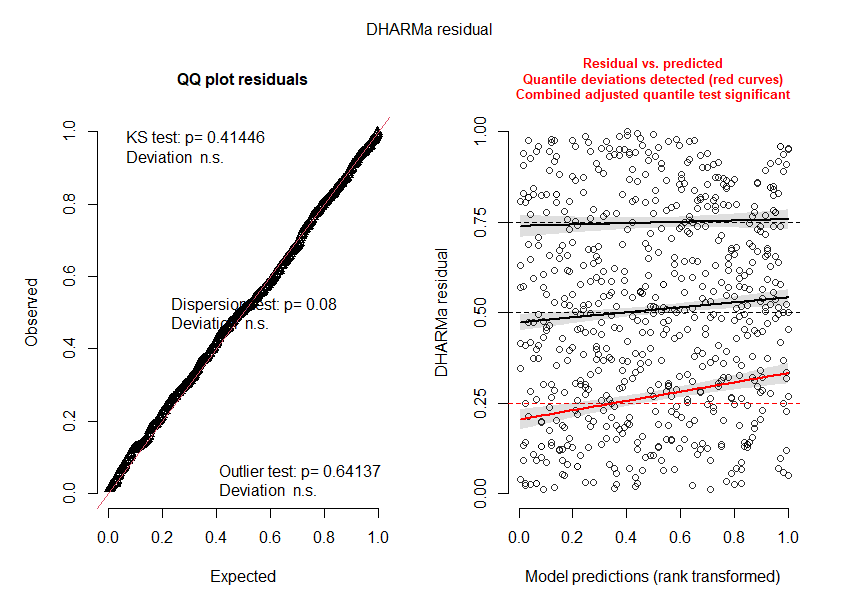


**Figure S3** Residual plots of the zero-inflated GLMM with a negative binomial distribution fitted with data on the variability in patch abundance. The QQ plot (left) shows there are no differences between observed and expected residuals. The residual plot (right) suggests that there are no differences detected at 0.5 and 0.75 quantiles, but there is a significant deviation at the 0.25 quantile.


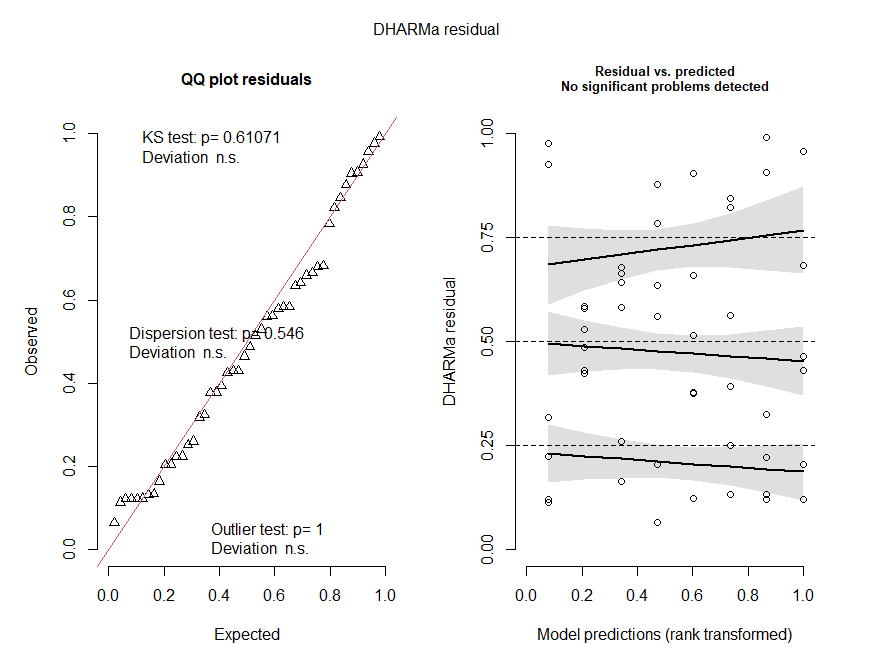


**Figure S4** Residual plots of the Gaussian GLM fitted with data on the maximum rate of extinction (log-transformed parameter b that was estimated from nonlinear regression). Both the QQ plot (left) and the residual plot (right) shows that there are no differences between observed and expected residuals.

**References:**

Hartig, F., & Hartig, M. F. (2017). Package ‘DHARMa’. R package.

Ritz, C., Baty, F., Streibig, J. C., & Gerhard, D. (2015). Dose-Response Analysis Using R. *Plos One, 10*(12). doi:10.1371/journal.pone.0146021
